# Hyposalivation Treatment with Non-Crosslinked Hyaluronic Acid and Amino Acids Solution—A Clinical Pilot Study

**DOI:** 10.3390/life16060887

**Published:** 2026-05-25

**Authors:** Marzena Liliana Wyganowska, Sylwia Klewin-Steinböck, Peng Ao

**Affiliations:** Department of Periodontology and Oral Mucosa Diseases, Poznan University of Medical Science, 70, Bukowska St., 60-812 Poznan, Poland; wyganowska@ump.edu.pl (M.L.W.); peng.ao@ump.edu.pl (P.A.)

**Keywords:** hyaluronic acid, amino acids, dry mouth, xerostomia

## Abstract

Background. This randomized controlled clinical study aimed to evaluate changes in salivary secretion following injections of a hyaluronic acid and amino acid solution in patients with hyposalivation. Materials and Methods. A total of 100 patients with symptoms of hyposalivation were randomly assigned to either a study group or a control group (1:1). The consistency and pH of unstimulated saliva, as well as the volume and buffering capacity of stimulated saliva, were assessed using the Saliva-Check Buffer GC test at baseline and after the study period. Patients in the study group received injections of a hyaluronic acid and amino acid solution into the buccal mucosa in four sessions at two-week intervals, while the control group received no intervention. Results: Significant improvements were observed in the intervention group, including favorable changes in the consistency and pH of unstimulated saliva as well as increases in the volume and buffering capacity of stimulated saliva over the study period. A paired *t*-test demonstrated a significant increase in salivary pH following treatment (t = −10.07, *p* < 0.001). The mean pH increased from 6.54 (95% CI: 6.42–6.65) at baseline to 7.31 (95% CI: 7.22–7.40) after the intervention, representing a mean increase of 0.78 units (95% CI: 0.62–0.93). Additionally, the Wilcoxon signed-rank test revealed a significant improvement in stimulated salivary flow (V = 1275, *p* < 0.001). The median salivary volume increased from 1.5 mL at baseline to 5.0 mL post-treatment, representing a median increase of 3.75 mL (95% CI: 3.5–4.0). No statistically significant changes were observed in the control group for saliva pH, consistency, or buffering capacity. Stimulated salivary flow showed a statistically significant difference (*p* = 0.002) but with a median increase of 0 mL, indicating no clinically meaningful change. Conclusions: Injections of a hyaluronic acid and amino acid solution may represent a promising therapeutic option for patients with symptoms of dry mouth. Study registered on Clinicaltrials NCT07309588.

## 1. Introduction

Dry mouth is one of the most common complaints among patients, in both dental and general medicine practices, especially among the older population. Xerostomia (or dry mouth) is a medical term used to describe the subjective sensation of dry mouth that commonly occurs because of decreased salivary flow (hyposalivation). Although the two terms do not correspond to the same conditions and should not be used interchangeably, many physicians do so, since most patients with xerostomia also have hyposalivation [1,2].

Approximately 30% of the population complains of some degree of dry mouth [3]. Occasionally, during dehydration or stress, it is not a cause for concern. The situation is more serious if it happens more often, such as every day. It could also point to an underlying health condition. In untreated patients, hyposalivation increases their vulnerability to tooth decay, gingivitis and oral mucosa sensation [4]. Dry mouth is related to pain, sometimes insomnia and psychological problems, and results in deterioration of life well-being.

Saliva is a hypotonic solution composed of 98.5% water with 1% organic and 0.5% inorganic components [5]. The organic and inorganic components of saliva are present in low concentrations, with some proteins synthesized in the gland (such as α-amylase) present in relatively high concentrations. Other organic components detected in saliva are vitamin C, maltase, urea, uric acid, lactase, hormones (testosterone, cortisol), albumin, creatinine, amino acids, mucin and immunoglobulins (IgA, IgG, IgM) [6]. Saliva α-amylase and lipase play a role in starch digestion and triglyceride decomposition. Salivary mucins lubricate intraoral structures and help to form a barrier against microbial invasion. Salivary immunoglobulins, especially secretory IgA (SIgA), play a key role in neutralizing toxins, agglutinating bacteria, and preventing their adhesion to mucosal surfaces [7].

By moistening the oral mucosa, saliva facilitates chewing, swallowing, and speech. Furthermore, a constant flow of saliva facilitates the mechanical removal of food debris and microorganisms and regulates the oral pH, protecting against acidic byproducts of bacterial metabolism. This buffering capacity is essential for maintaining a healthy oral microbiome and preventing demineralization of tooth enamel. Saliva also serves as a protective factor against infections because of its numerous organic components. People with dry mouth not only have trouble chewing and swallowing food but also have difficulty tasting and speaking, and a reduced tolerance for dentures. Furthermore, xerostomia increases the risk of caries, periodontal disease, candidiasis, oral ulcers, and dysphagia, which can negatively impact nutritional status and quality of life.

There is wide variability in individual salivary flow rates. The average daily flow of whole saliva varies between 0.5 and 1.5 L. The accepted range for normal unstimulated salivary flow is above 0.1 mL/min. Any unstimulated flow rate below 0.1 mL/min is considered hypofunction [3]. Stimulated saliva represents 80 to 90% of daily salivary production, and the stimulated flow rate varies from 1 to 3 mL/minute [8].

Saliva is produced by major salivary glands (MaSGs)—the parotid, submandibular, and sublingual glands—and by approximately 600 to 1000 minor salivary glands (MiSGs) located in the submucosal layer of the oral cavity [9]. The major salivary glands contribute approximately 90% of total saliva secretion, whereas MiSGs account for the remaining 10%. Minor salivary glands and the sublingual glands are responsible for lubrication and secrete saliva continuously, including during the night, thereby contributing mainly to resting salivary flow, whereas the parotid and submandibular glands produce saliva predominantly in response to stimulation, such as mastication or gustatory stimuli [10]. MiSGs are important for preventing the subjective sensation of dry mouth, since the saliva produced by these glands creates a lubricating micron-thick film that protects the oral mucosa. Furthermore, MiSGs play a key role in saliva production during sleep; therefore, reduced MiSG flow appears to be a cause of nocturnal dry mouth [9]. Damage to the minor salivary glands leads to a loss of local protection, manifested by increased susceptibility to chemical stimuli, microtrauma, inflammation, and reduced secretion of saliva containing mucins, IgA, defensins, EGF, and factors responsible for epithelial surface lubrication [11].

Hyaluronic acid (HA) may exert a protective effect on the microenvironment of the minor salivary glands by reducing inflammatory infiltration in the periglandular tissue, improving conditions for the regeneration of secretory cells, and protecting against keratinization and obstruction of the excretory ducts. HA is an important component of the extracellular matrix (ECM) during salivary gland development. Condensation of HA around progenitor cells has been demonstrated, along with its influence on CD44 receptors on the cell membrane of minor salivary glands [12,13]. Interactions between hyaluronic acid (HA) and CD44, as well as interactions of CD44 with the actin cytoskeleton and multiple growth factor receptors, are key regulators of communication between the cell and its microenvironment [14].

Amino acids may exhibit a supportive role in the protection of the oral mucosa and salivary glands. Their protective effects have been demonstrated during anticancer therapy and under conditions of cellular stress; the underlying mechanisms include support of cellular metabolism, maintenance of barrier function, and reduction in tissue damage [15]. Dysregulation of amino acid metabolism in saliva (salivary metabolomics) has also been suggested to play a role in the pathophysiology of dryness [16].

Despite the various etiological factors underlying xerostomia/hyposalivation, the available treatment options do not differ because of the cause and are predominantly symptomatic—except in cases of drug-induced xerostomia, where therapy involves discontinuation of the systemic medication. Therapeutic strategies implemented to manage xerostomia, regardless of its etiology, have thus far failed to yield definitive or consistently effective outcomes.

The rationale for intramucosal administration of HA and AAs was partially extrapolated from aesthetic medicine, where intradermal HA-based biorevitalization and mesotherapy are widely used to improve tissue hydration, elasticity, microcirculation, and regenerative activity through prolonged local effects after injection. In contrast to topical formulations, intramucosal injection may provide a local depot effect and prolonged interaction with surrounding tissues, including minor salivary glands and the oral mucosa, potentially enhancing hydration and tissue regeneration. Lysine, proline, leucine, valine, and alanine are involved in the regenerative and metabolic processes of minor salivary glands by influencing protein synthesis, connective tissue remodeling, and the response to oxidative stress. In particular, proline and lysine are associated with collagen metabolism and tissue repair, whereas leucine and valine support the proliferation and metabolic activity of secretory cells through pathways related to protein synthesis. Salivary metabolomic studies have demonstrated altered concentrations of these amino acids in diseases associated with minor salivary gland dysfunction, such as Sjögren syndrome, suggesting their involvement in inflammatory and regenerative processes within salivary glands [17,18].

The aim of this study was to evaluate changes in saliva quality (flow, pH, and buffer) and individual patients’ perception of quality of life following the injection of a solution of low-molecular-weight hyaluronic acid (HA) and amino acids (AAs) into the buccal mucosa of patients with hyposalivation.

## 2. Materials and Methods

### 2.1. Ethical Statement

This study was conducted in accordance with the ethical principles of the Declaration of Helsinki and was approved by the Bioethics Committee of Poznan University of Medical Sciences (Resolution No. 526/24). The study was registered at ClinicalTrials.gov (NCT07309588) on 29 December 2025. Written informed consent was obtained from all participants prior to participation. This study follows the CONSORT 2025 checklist: guideline for reporting randomized trials (Supplementary Materials).

### 2.2. Material

This study used a class III injectable medical device (CE Certificate no. QCT-0153-20 add. 107-21, EPG-0384-21) containing a mixture of low-molecular hyaluronic acid (HA) and specific amino acids (AAs) (glycine, L-proline, L-leucine, L-lysine, L-valine and L-alanine).

### 2.3. Study Population

**Figure 1:**
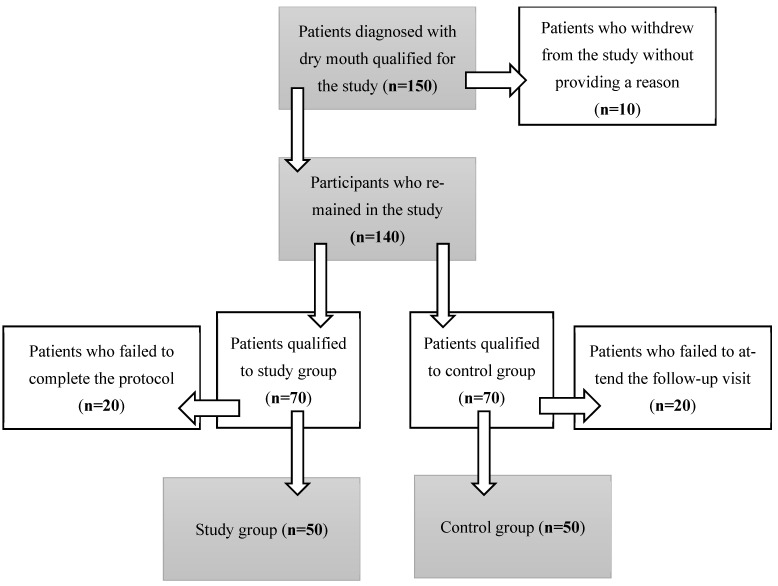
Study population.

The study was designed as a pilot interventional study evaluating the effect of an intervention on the severity of xerostomia among adult residents of Poznan aged 38–78 years. Only individuals with pre-existing symptoms of dry mouth (xerostomia) who met the inclusion criteria were enrolled. One hundred and fifty patients with dry mouth syndrome were screened; of these, 100 met the eligibility criteria and were enrolled. Participants were randomly assigned in a 1:1 ratio to either the intervention group (n = 50) or the control group (n = 50) using computer-generated stratified randomization based on age and sex to ensure balanced baseline characteristics between groups (Figure 1). Due to the pilot nature of the study and the limited sample size, block randomization and formal allocation concealment procedures were not applied. The sample size was determined pragmatically, in line with the objectives of a pilot study. Given the pilot nature of the study, comparisons of changes between the intervention and control groups are exploratory, and the study is not powered to detect small between-group differences with high statistical power. The results will be used to inform the design and formal sample size calculation of a subsequent full-scale study.

The study was conducted in a group of patients of both sexes treated at the Department of Periodontology and Oral Mucosa Diseases, Poznan University of Medical Sciences. The main complaint reported by patients included in the study was a subjective feeling of dry mouth related to reduced salivary secretion. Additional symptoms included difficulties with eating, speaking, or swallowing; taste disturbances; problems with wearing dentures; a sensation of the tongue adhering to the palate; halitosis; insomnia and pain. Many patients reported nocturnal xerostomia, which made falling asleep difficult or impossible.

The exclusion criteria were smoking, pregnancy or breastfeeding, diabetes mellitus, immunosuppressive therapy, neoplastic diseases, and current or previous radiotherapy for head and neck cancer. Sjögren’s syndrome and active inflammatory conditions of the oral cavity were also excluded. Patients with other concomitant systemic diseases, such as hypertension, hypothyroidism, or asthma, as well as patients receiving antidepressant therapy, were eligible for inclusion.

### 2.4. Study Design

Prior to the saliva test, the participants were instructed to refrain from caffeine and alcohol for at least 24 h. Additionally, patients were instructed to abstain from eating, drinking, and brushing their teeth before the collection of saliva samples. Saliva samples from all patients were collected between 8:00 A.M. and 10:00 A.M. Ten minutes before saliva collection, patients rinsed their mouths with water.

The patients completed an auctorial questionnaire before treatment (Table 1). The pain level was measured with VAS. Patients also completed an intraoral examination, including a “mirror test” to evaluate unstimulated saliva flow. To evaluate both unstimulated and stimulated saliva characteristics, a Saliva-Check Buffer GC test kit was used.

The Visual Analogue Scale (VAS) allows for the assessment of pain intensity on a scale from 0 to 10, where 0 indicates no pain; 1–3—indicates mild pain; 4–6—indicates moderate pain; 7–9—indicates severe pain; and 10—indicates unbearable pain. The percentage of patients who achieve a 30% reduction in pain will be considered a clinically meaningful improvement, while a 50% reduction will be regarded as a clinical success.

During the “mirror test”, a dental mirror was gently moved along the buccal mucosa to observe how easily the mirror moved. A lack of resistance or easy movement suggested normal saliva production, increased resistance, or a “sticking” sensation, indicating reduced saliva production.

The consistency of unstimulated saliva was assessed visually by the same dentist, examining the resting saliva in the oral cavity. The sticky frothy saliva residues indicated increased viscosity, the frothy bubbly saliva residues indicated increased viscosity and the watery clear saliva residues indicated normal viscosity. These three categories are referred to in the Results as SSIV (sticky saliva with increased viscosity), BSIV (bubbly saliva with increased viscosity), and WSNV (watery saliva with normal viscosity).

The pH measurement was performed by placing a pH test strip in a sample of unstimulated saliva for 10 s and comparing the results with the test chart included in the package. The samples were divided into strongly acidic (pH 5.0–5.8), moderately acidic (pH 6.0–6.6) and healthy saliva (pH 6.8–7.8) groups.

Two tests of stimulated saliva were performed: quantity and buffering capacity. The patient was instructed to chew a piece of wax to stimulate saliva production. After 30 s, the patient expectorated saliva into a spittoon. Chewing continued for another 5 min, and saliva was collected in a container at regular intervals. Saliva volume was measured by checking the ml markings on the container wall.

The test strip included in the kit was used to test buffering capacity. One drop of stimulated saliva was pipetted onto each of the three test patches. Excess saliva was removed with absorbent tissue to prevent it from affecting the accuracy of the test results. The result was calculated after 2 min according to the conversion table provided in the kit.

The same complete set of tests and questionnaires was repeated two weeks after treatment finished (and after eight weeks in the control group).

Stimulated saliva flow and patient-reported xerostomia-related discomfort assessed using the VAS were defined as the primary outcomes of the study. The remaining evaluated parameters, including the mirror test, unstimulated saliva consistency, unstimulated saliva pH, and stimulated saliva buffering capacity, were treated as secondary outcomes.

In the control and study groups, patients were asked to discontinue the use of mucosa lubricants, except for drinking water. Patients were also asked not to change their daily oral hygiene protocol. A drawback of the study was the lack of a placebo group; – this was a preliminary clinical trial.

### 2.5. Intervention

**Figure 2:**
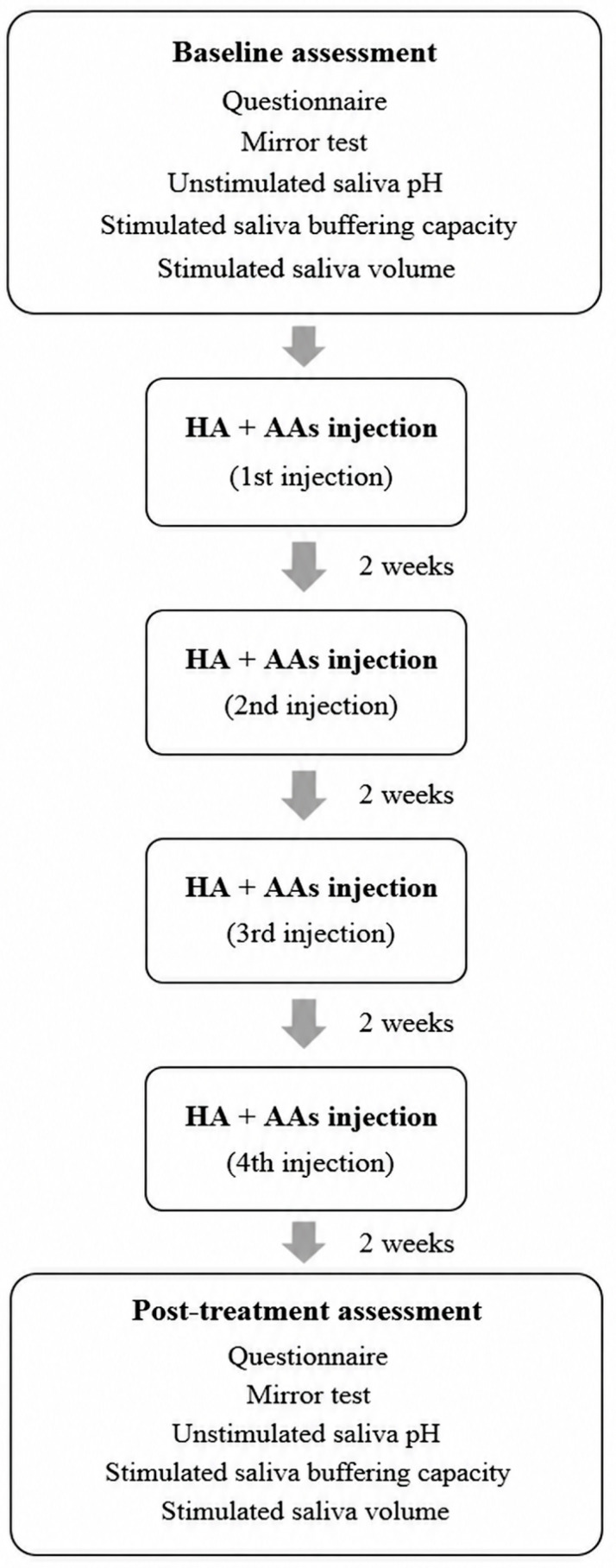
Clinical trial design for the study group.

**Figure 3:**
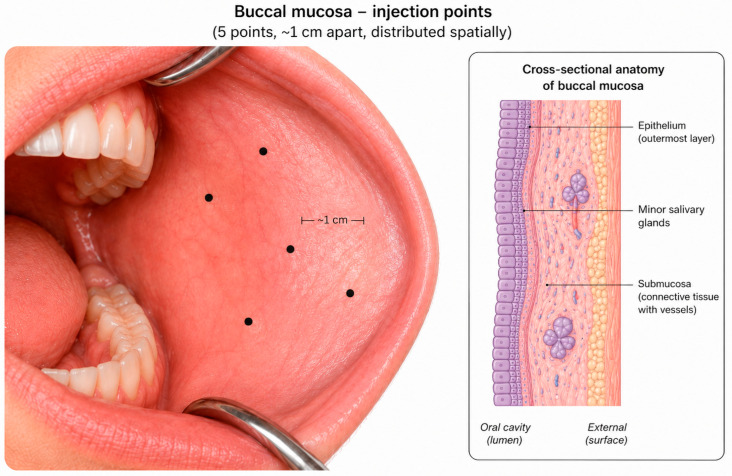
A schematic diagram of the anatomical distribution of injection sites on the buccal mucosa (Source: Author’s contribution).

In the study group, HA and AA solutions were used. A total of 2 mL (1 mL on each side) of solution was injected into the buccal mucosa four times at two-week intervals (Figure 2). The distance between the points of the injections was approximately 10 mm. The total number of injection points per side was 5 (Figure 3). Cosmetic syringes (1 mL) and needles 30 G 4 mm were used for injection. Any adverse effects reported by participants throughout the intervention period were recorded in their medical charts.

### 2.6. Statistical Analysis

All analyses were conducted in R (v4.4.2; R Foundation for Statistical Computing, Vienna, Austria) within the RStudio IDE version 2025.09 (Posit Software, PBC, Boston, MA, USA). Descriptive statistics were used to summarize all the variables. We assessed the normality of continuous measures via the Shapiro–Wilk and Anderson–Darling tests to ensure robustness. For inferential comparisons of paired data, we selected tests according to distributional assumptions: normally distributed variables were analyzed with paired *t*-tests, whereas salivary pH, stimulated salivary flow rate, and buffering capacity—showing marked deviations from normality—were examined via the Wilcoxon signed-rank test. Changes in the ordinal variable saliva consistency (SSIV vs. BSIV) were evaluated via McNemar’s test. The significance level was set at α = 0.05. The Wilson score method was used to calculate 95% confidence intervals for proportions, which works well when sample sizes are small or proportions are near 0 or 1. Exact (Clopper–Pearson) intervals gave nearly identical results. All *p*-values are reported to three decimal places, with values below 0.001 reported as *p* < 0.001. All visualizations—including histograms of pH changes, Sankey diagrams for consistency transitions, boxplots of salivary flow, and figures for buffering capacity—were generated programmatically via R packages.

## 3. Results

### 3.1. Demographic and Health-Related Analysis

The demographic and health-related characteristics of the participants are shown in Table 2. A predominance of female participants was observed (72% in the study group and 66% in the control group). The most common systemic disease was hypertension (77% in the study group and 56% in the control group).

The symptom characteristics of participants based on the patient questionnaire obtained before and after treatment are shown in Table 3. The predominant symptoms in both groups were difficulty swallowing food (92% and 86%, respectively) and feeling a dry mouth after waking up (92% and 90%, respectively).

### 3.2. VAS

The VAS test results are presented in Table 4. At the beginning of the clinical trial, most patients reported moderate pain (50% in the study group and 52% in the control group) or severe pain (44% in the study group and 40% in the control group). At the end of the clinical trial, most patients in the study group reported no pain (52%) or mild pain (40%), indicating clinical success. In the control group, there was a significant increase in the number of patients reporting unbearable pain (from 2% to 12%), which may be due to the discontinuation of saliva stimulants.

### 3.3. Stimulated Saliva Flow

**Figure 4:**
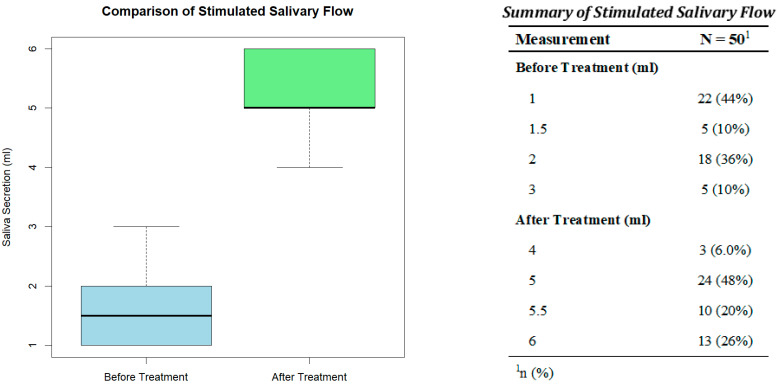
Boxplots of stimulated salivary flow (mL) before and after treatment in the study group. The y-axis shows volume in mL; the x-axis shows the time point (before/after).

There were statistically significant differences in stimulated salivary flow rates between pre- and post-treatment conditions in the study group, as shown in Figure 4 and Table 5. After treatment, the stimulated salivary flow had a greater mean (5.30 mL) and median (5.0 mL) than the pre-treatment levels did in the study group.

The analysis revealed that stimulated salivary flow did not follow a normal distribution under either condition, as indicated by positive kurtosis values greater than 0. Following treatment, the mean flow rate increased to 5.30 mL, with a corresponding median of 5.0 mL, compared with lower pre-treatment values.

The results from the Wilcoxon signed-rank test (Table 6) confirmed a statistically significant improvement in salivary flow (V = 1275, *p* < 0.001). The median value rose from 1.5 mL at baseline to 5.0 mL post-treatment, reflecting a median shift of 3.75 mL (95% CI [3.5, 4.0]). This substantial increase suggests both statistical and clinical relevance.

In the control group, no significant changes were observed during the study period. In 5 of the 50 patients (10%), the saliva volume was slightly reduced (Table 7).

**Figure 5:**
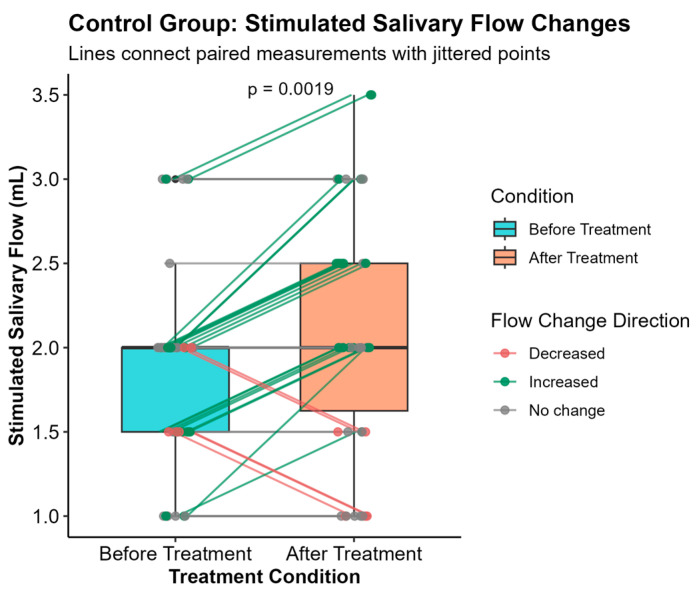
Boxplots of stimulated salivary flow (mL) before and after the study period in the control group. The y-axis shows volume in mL; the x-axis shows the time point (before/after).

The mean stimulated salivary flow showed a modest increase, from 1.88 mL at baseline to 2.07 mL after the study period. Normality testing indicated clear deviations from a normal distribution. The Shapiro–Wilk test produced *p*-values of <0.0001 before treatment and 0.0017 after the research period, whereas the Anderson–Darling test gave *p* < 0.0001 and *p* = 0.0002, respectively (Table 8). As all values fell below the 0.05 threshold, the assumption of normality was not supported. On this basis, the nonparametric Wilcoxon signed-rank test was used to compare paired measurements of stimulated salivary flow before and after treatment (Figure 5).

A Wilcoxon signed-rank test was conducted to compare paired measurements. The analysis yielded V = 270, *p* = 0.0019, indicating a difference between pre- and post-observation values. The median difference was 0 mL (95% CI: 0 to 0.5 mL). The effect size (r = 0.439) suggested a moderate magnitude of change in stimulated salivary flow following the observation time in the control group. Changes in stimulated salivary flow may be caused by increased or decreased water intake, patient well-being on a given day or external factors. Despite the statistically significant *p*-value, the median difference of 0 mL and the small mean increase (from 1.88 to 2.07 mL) suggest that this change is unlikely to be clinically meaningful.

### 3.4. Unstimulated Saliva Test—“Mirror Test”

**Figure 6:**
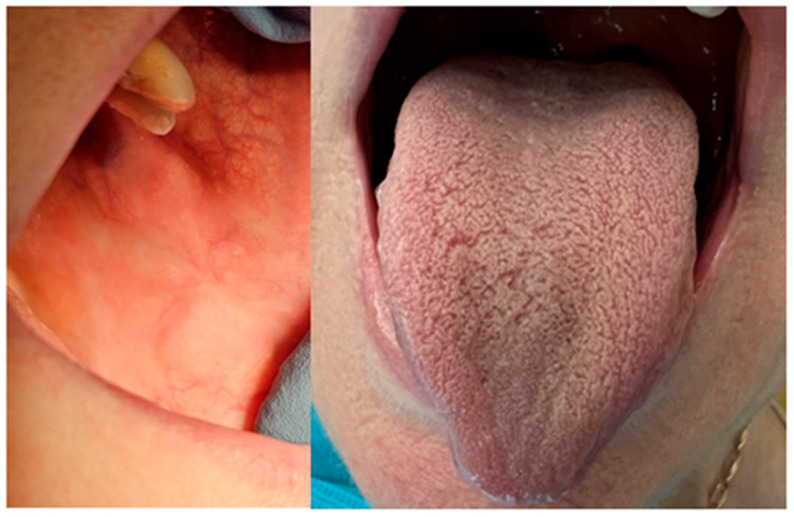
Example of buccal and tongue mucosa—before treatment—study group.

**Figure 7:**
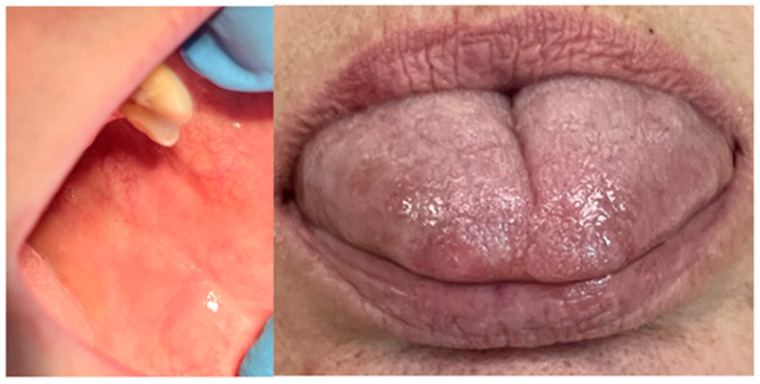
Example of buccal and tongue mucosa in the same patient—after treatment—study group.

Before treatment, during the intraoral examination, the buccal mucosa, tongue and lips were visually assessed. The mucous membrane was dull and dry (Figure 6). A “mirror test” was performed. In 100% of the patients (in both groups), the mirror did not move after contact with the buccal mucosa or moved with difficulty, causing discomfort to the patient. Two weeks after the end of the treatment, the mucous membrane in the study group was visibly moisturized (Figure 7). One hundred percent of 50 patients in the study group improved during clinical examination, at which point the examination mirror changed from non-movable to movable. In contrast, in the control group, the mirror was unmovable in contact with the mucosa.

### 3.5. Unstimulated Saliva Test—pH Value

The saliva pH at the beginning of the study was within the range of 5.2–6.8 in the control group and 5.2–6.4 in the study group, which indicates highly or moderately acidic saliva in most patients. The analysis compared pre- and post-treatment measurements to evaluate treatment efficacy.

**Figure 8:**
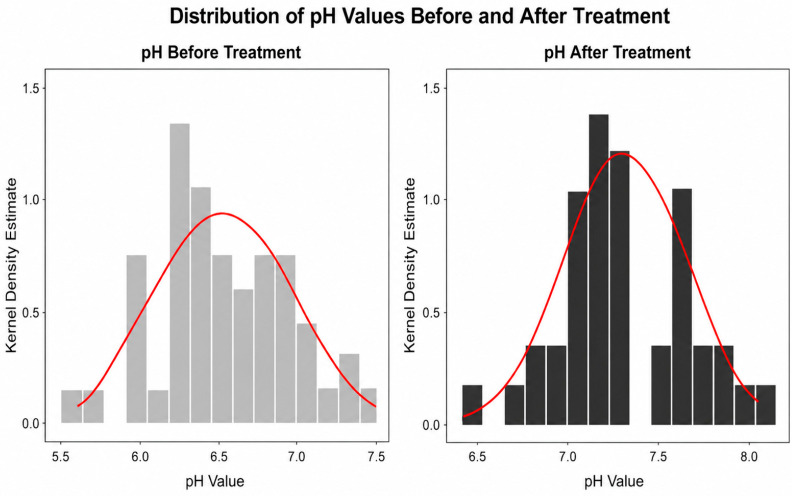
Kernel density estimates of salivary pH before and after treatment in the study group. The y-axis shows kernel density; the x-axis shows pH units.

Significant changes were observed in the study group. Salivary pH ranged from 6.2 to 7.6, with 82% of patients presenting a pH ≥ 6.8, which is indicative of healthy saliva (Table 9, Figure 8).

Salivary pH values were determined via the Shapiro–Wilk test before treatment (W = 0.989, *p* = 0.928) and after treatment (W = 0.991, *p* = 0.962), from which it can be concluded that there is no significant evidence to suggest that the data deviate from normality. Therefore, a parametric paired *t*-test is adapted to analyze the mean difference between these two variables (Table 10).

A paired *t*-test revealed a significant increase in salivary pH following treatment (t = −10.07, *p* < 0.001). The mean value rose from 6.54 (95% CI: 6.42–6.65) at baseline to 7.31 (95% CI: 7.22–7.40) after the intervention, yielding a mean difference of −0.78 units (95% CI: −0.93 to −0.62). The t-statistic is negative because subtraction was performed as baseline minus post-treatment; the increase is 0.78 units (95% CI: 0.62–0.93). Although the negative sign reflects test directionality, the results clearly demonstrate a shift from acidic (pH < 7.0) to neutral levels (pH > 7.0). This normalization of the oral pH suggests that this treatment may help protect against enamel erosion and mucosal irritation.

**Figure 9:**
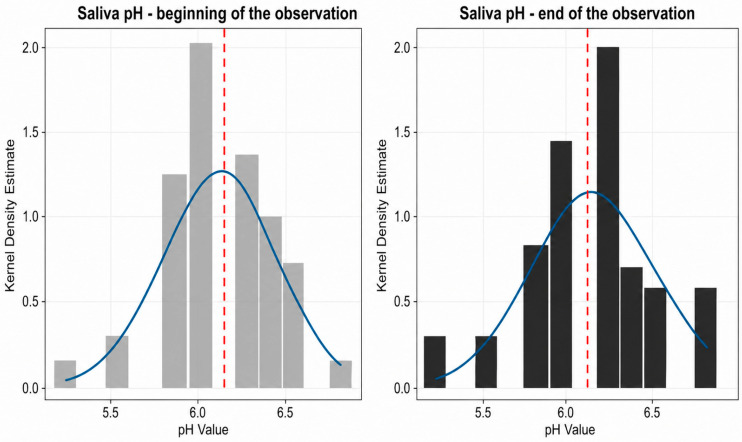
Kernel density estimates of salivary pH before and after the study period in the control group. The y-axis shows kernel density; the x-axis shows pH units.

During the follow-up visit after 8 weeks, no changes in saliva pH were observed in patients in the control group (Table 11, Figure 9).

The Shapiro–Wilk test indicated that the salivary pH values deviated from a normal distribution both before (W = 0.939, *p* = 0.012) and after the study period (W = 0.951, *p* = 0.039). Since both results were significant at the 0.05 level, the assumption of normality was not satisfied. Consequently, the Wilcoxon signed-rank test was used to assess paired differences in pH measurements.

Analysis of paired salivary pH values in 50 patients revealed no significant changes across the study period. The Wilcoxon signed-rank test yielded V = 94.5 with *p* = 0.095, which is above the 0.05 threshold for significance. The mean difference was −0.04 pH units (95% CI: −0.09 to 0.01), suggesting a slight downward trend. However, as the confidence interval crosses zero, the observed variation is unlikely to reflect a clinically meaningful shift.

### 3.6. Unstimulated Saliva Test—Consistency

There were significant changes in saliva consistency in the study group. Initially, most patients (92%) were characterized by sticky saliva with increased viscosity (SSIV, corresponding to the “sticky frothy” appearance described in Methods). (Table 12 and Table 13). After treatment, most of the patients (80%) were characterized by watery saliva with normal viscosity (WSNV).

The analysis demonstrated a marked improvement in saliva consistency after treatment (McNemar’s test, *p* < 0.001). At baseline, 46 patients (92.0%; 95% CI: 85.1–98.9%) presented with sticky saliva of increased viscosity (SSIV), whereas 4 patients (8.0%; 95% CI: 3.2–18.8% presented with bubbly saliva with increased viscosity (BSIV). Following treatment, SSIV resolved completely, and 40 patients (80.0%; 95% CI: 69.1–90.9%) attained normal watery saliva, whereas 10 patients (20.0%; 95% CI: 9.1–30.9%) continued to present with BSIV. This shift reflects a substantial clinical improvement, with four-fifths of the cohort achieving normal viscosity after intervention.

**Figure 10:**
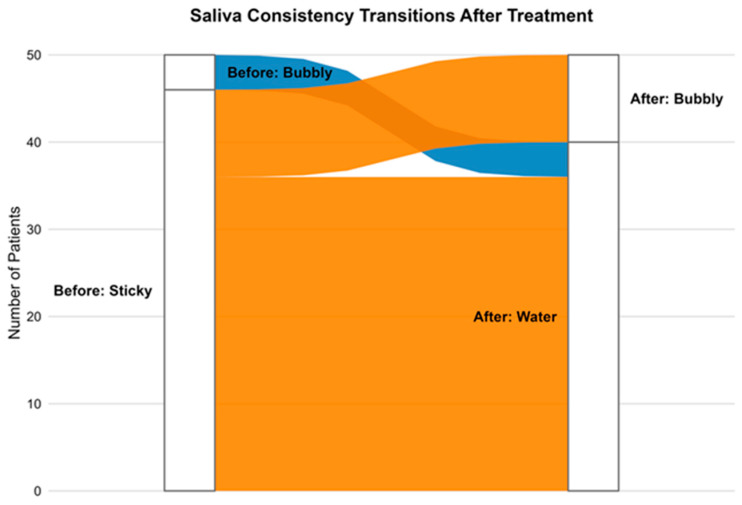
Saliva consistency transition—study group.

The Sankey diagram (Figure 10) illustrates changes in saliva viscosity following treatment. Among the 46 patients with sticky saliva and increased viscosity (SSIV) at baseline, 36 (78.3%; 95% CI: 66.4–87.2%) achieved normal watery saliva (WSNV), reflecting marked improvement. Ten SSIV patients (21.7%; 95% CI: 12.8–33.6%) shifted to bubbly saliva with increased viscosity (BSIV). All four baseline BSIV cases (100%; 95% CI: 51.0–100%) converted to WSNV. Notably, no patients retained SSIV after treatment (0/50; 0%; 95% CI: 0–7.1%), indicating complete resolution of the sticky saliva state and a strong therapeutic effect on viscosity normalization.

At the beginning of the study period, 39 patients (78%) exhibited SSIV, and 11 patients (22%) exhibited BSIV. These proportions remained unchanged after treatment (Table 14 and Table 15).

**Figure 11:**
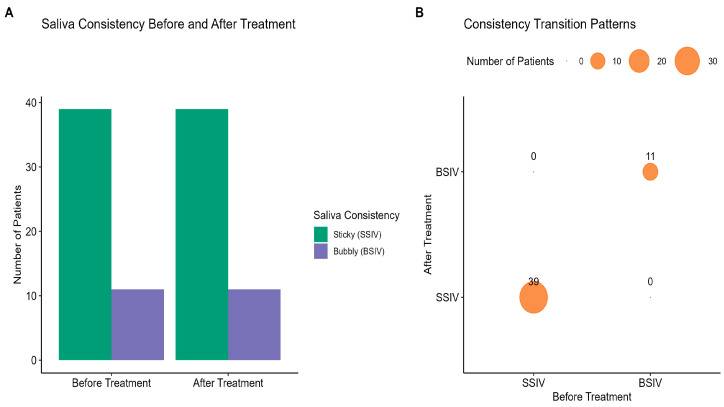
Control group saliva consistency. (**A**) Bar chart showing the number of patients with each consistency category before and after the study period (y-axis: number of patients; x-axis: consistency category). (**B**) Transition patterns between categories (no changes observed).

To evaluate changes in saliva consistency, McNemar’s test would normally be used for paired nominal data. However, because no patient transitioned between consistency categories (SSIV and BSIV), McNemar’s test is not informative; there were zero discordant pairs. By simple inspection of Table 15 and Figure 11, there was no change in saliva consistency in the control group over the study period.

### 3.7. Stimulated Saliva—Buffering Capacity

Buffering capacity is a fundamental property of saliva, maintaining oral pH stability and protecting against acid-induced demineralization and microbial imbalance. It serves as a critical physiological and biochemical marker for oral health, with implications for the prevention and management of xerostomia, dental caries, and periodontal disease. Monitoring variations in this parameter offers insight into treatment efficacy and preventive strategies, reinforcing its importance in diagnostic assessment and biomarker research.

**Figure 12:**
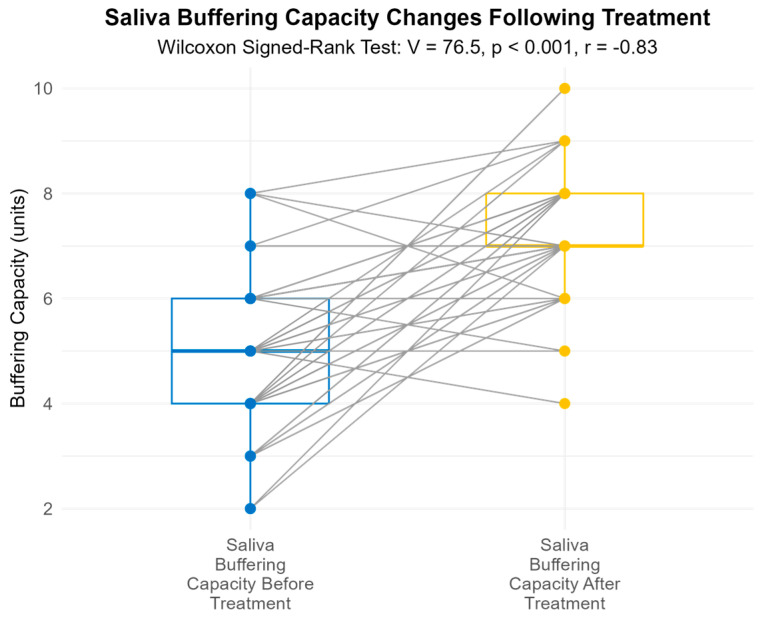
Boxplots of saliva buffering capacity (score, range 0–12) before and after treatment in the study group. The y-axis shows buffering capacity score; the x-axis shows time point (before/after).

Shapiro–Wilk tests revealed nonnormal distributions for both pre-treatment (W = 0.926, *p* = 0.004) and post-treatment (W = 0.948, *p* = 0.028) buffering capacity scores (Table 16 and Table 17, Figure 12). Thus, nonparametric Wilcoxon signed-rank tests were used for analysis.

**Figure 13:**
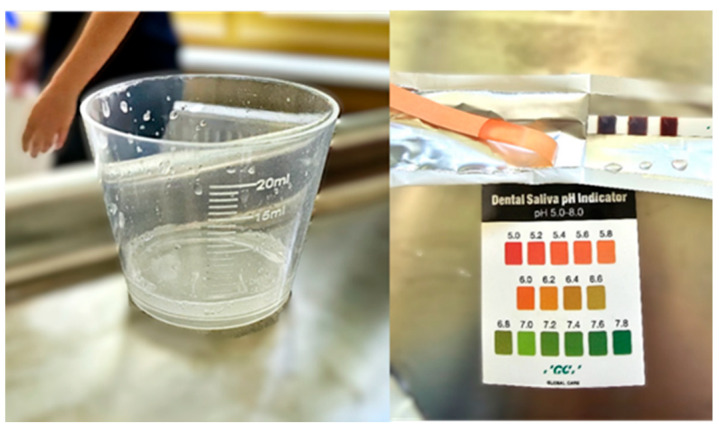
Saliva-Check Buffer GC (stimulated saliva volume and buffering capacity, unstimulated saliva pH)—results before treatment.

**Figure 14:**
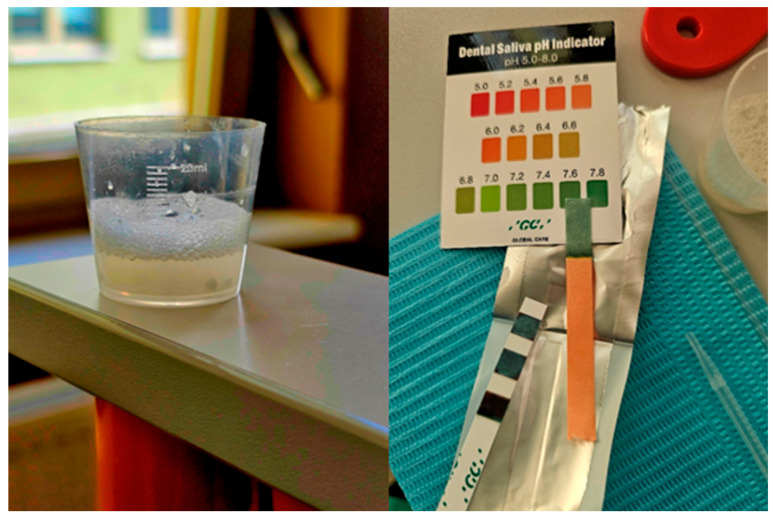
Saliva-Check Buffer GC (stimulated saliva volume and buffering capacity, unstimulated saliva pH)—results after treatment.

The analysis revealed a significant improvement in saliva buffering capacity after treatment (Wilcoxon V = 76.5, *p* < 0.001). The effect size (r = −0.83) reflected a large shift in values, with the post-treatment buffering capacity markedly higher than the baseline buffering capacity (Table 18). This outcome indicates that the treatment protocol was effective in enhancing salivary function in patients with xerostomia. The upward trends observed across individuals, despite differences in baseline levels, support the reliability of the intervention (Figure 13 and Figure 14).

No significant changes were observed in the control group during the study period. In the control group, the same mean salivary buffering capacity of 4.72 was observed (Table 19). Both before and after the study period, the buffering capacity data fail the normality assumption according to both the Shapiro–Wilk test and the Anderson–Darling test (very small *p* values < 0.05) (Table 20). Therefore, the nonparametric Wilcoxon signed-rank test was used to analyze differences in salivary buffering capacity before and after the study.

**Figure 15:**
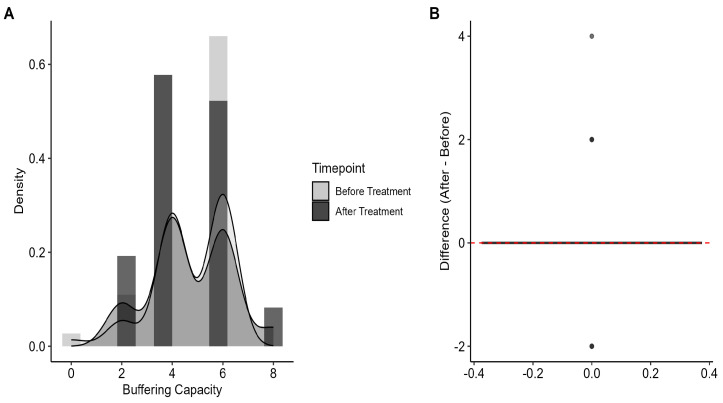
Control group buffering capacity. (**A**) Histogram (or kernel density) of buffering capacity scores before (light grey) and after (dark grey); y-axis: frequency (or kernel density), x-axis: buffering capacity score. (**B**) Boxplot of paired differences (after minus before); y-axis: difference in score, horizontal line at zero indicates no change.

Assessment of the data distribution with the Shapiro–Wilk and Anderson–Darling tests revealed significant deviations from normality at both baseline and after the study period. At baseline, the *p*-values were 1.35 × 10^−7^ (Shapiro–Wilk) and 2.28 × 10^−12^ (Anderson–Darling), whereas the post-observation values were 3.34 × 10^−5^ and 3.34 × 10^−8^, respectively (Table 20). Because the assumption of normality was violated, the Wilcoxon signed-rank test was applied to compare paired values. The test yielded V = 45.5 with *p* = 1, and the rank-biserial correlation was 0. These results indicate that there was no detectable change in salivary buffering capacity within the control group (Figure 15).

## 4. Discussion

A lack of saliva can be devastating for patients’ oral health and can reduce their daily quality of life. The exact occurrence of hyposalivation or xerostomia in the population is unknown. Studies have shown an increase in prevalence with age as well as gender-dependent prevalence. In the general population, the prevalence ranges from 5.5% to 46% [4], with 30% in adults over 65 years of age and 40% in adults over 80 years of age [20]. It makes xerostomia one of the most common oral problems. Dry mouth remains an unresolved common complaint, especially among the geriatric population, despite seeking medical or dental help.

Dry mouth syndrome can be observed without an actual reduction in salivary flow [1]. The other cause is objectively assessed salivary gland hypofunction, which could be attributed to several systemic diseases, such as Sjögren syndrome, rheumatoid arthritis, systemic lupus erythematosus, and medications used for systemic disease treatment. Addictions, such as smoking, alcohol use, caffeinated drinks, and some mouthwash, can cause oral dryness [21]. Current studies show that the most common cause of dry mouth is a side effect of various medications [22]. A review of the most frequently prescribed drugs revealed that the most common oral side effects are dry mouth (80.5%), dysgeusia (47.5%), and stomatitis (33.9%) [21]. The drugs most often causing dry mouth are tricyclic antidepressants, antipsychotics, atropines, beta-blockers and antihistamines [23]. In the case of the issue being caused by pharmacotherapy, the severity of dry mouth is correlated with the dose and number of medications taken. Polytherapy and polypharmacy are the most common causes of salivary gland hypofunction and xerostomia in the geriatric population. In patients with drug-induced xerostomia, medication changes may be considered. Several studies have shown improvement in 41% of patients who changed medications [24]; however, this improvement is not always possible in clinical practice. Hyposalivation is also an adverse effect of head and neck radiotherapy and occurs in 80% of patients. Its occurrence is dose-dependent and often irreversible.

Hyposalivation is more common in women, occurring significantly more often in perimenopausal and postmenopausal women (43%) than in premenopausal women (6%) [25]. Menopause is a normal stage of the female lifecycle marked by biological and hormonal changes. Because the oral mucosa and salivary glands contain estrogen receptors [26], changes in hormonal levels directly impact the oral cavity. Furthermore, salivary flow depends upon estrogen levels and estrogen deficiency can lead to a reduction in salivary flow [27]. A study by Minicucci et al. revealed a decrease in salivary secretion in a group of menopausal women compared with premenopausal women [28].

Several studies have considered changes in saliva quantity and quality throughout life. No consensus has been reached on the decrease in the salivary flow rate with age. Despite this, recent research by Vandenberghe-Descamps et al. [29] revealed a 38.5% reduction in the resting salivary flow rate and a 38.0% reduction in the stimulated salivary flow rate in elderly subjects compared with young subjects. The cause of salivary flow decline has been connected to the loss of acinar cells, loss of secretory tissue and increased adiposity, as well as neurophysiological deterioration [30]. Additionally, changes in saliva composition with age have been reported in some studies. Most notably, the mucin concentration decreases, which promotes the development of diseases of the oral mucosa and dry mouth [31].

The prevalence of hyposalivation and its consequences are increasing due to population aging, the effects of some systemic diseases, medical management, and commonly prescribed medications that reduce saliva production. Despite the significant occurrence of xerostomia in the general population, no standard treatment guidelines have been developed.

The treatment of dry mouth is difficult and long-term. It includes causal and symptomatic treatment as well as patient education. The aim of the therapy is to reduce patient discomfort and prevent complications, including caries and periodontal disease.

The dietary recommendations include drinking 1.5–2 L of water daily, avoiding dense and dry foods, cutting meals into small portions, chewing well before swallowing and drinking water during mealtime. It is also recommended to limit the consumption of alcohol, coffee, sweetened beverages and sweets, as well as hard, spicy foods irritating mucous membranes [1]. If applicable, patients should immediately stop smoking.

Currently, therapies for dry mouth can be divided into three groups: salivary stimulants, symptomatic treatments and regenerative and gene therapies.

Pharmaceutical treatment is intended to stimulate the secretion of water, electrolytes and macromolecules [32]. The most used are parasympathomimetic, such as pilocarpine [33], cevimeline and bethanechol. These substances can increase the secretion of saliva and relieve symptoms of xerostomia induced by radiotherapy or Sjogren syndrome. However, they may also cause adverse cardiovascular and pulmonary effects and/or nausea and dizziness.

Several authors [25,27] have shown that postmenopausal women experience relief from oral discomfort after hormone replacement therapy. Salivary estradiol levels returned to normal after hormone replacement therapy. Consequently, the corresponding symptoms of dry mouth also subsided.

Increasing fluid intake may temporarily relieve dry mouth, but other functions of saliva, such as coating and lubrication, cannot be achieved in this way. Some mouthwashes or oral sprays are used to relieve symptoms, but their effects are short-term. Research is limited, but the results suggest that a topical spray containing 1% malic acid may be effective in treating xerostomia [34].

Constant attempts are being made to find, if not effective, at least a more effective method for treating xerostomia. Research has been conducted on the use of fat tissue grafts for xerostomia treatment based on evidence that adipose-derived stem cells (ASCs) can differentiate into salivary gland cells [35]. Some studies indicate the potential usefulness of gene therapies in protecting salivary glands from ionizing radiation and preventing xerostomia [36]. Several clinical trials using hyperbaric oxygen therapy have shown promising results in treating dry mouth. The limited number or lack of hyperbaric chambers in many countries is a limitation of this method [37].

Our research is pioneering in this field. There is no possibility to compare our research with other studies because of the lack of similar studies. There are publications on the effects of cross-linked hyaluronic acid [38,39] and atelocollagen [40,41] on gingival tissue. None of the previously mentioned studies investigated the effects of these substances on salivary secretion and mouth dryness. Low-molecular-weight HA and AA solutions are used in skin rejuvenation and hydration in aesthetic medicine. Hyaluronic acid plays an important role in skin hydration because of its high ability to attract water molecules. Depending on the study, the results show that skin hydration increased by 25.9% 30 days after the last session and by 15.9% 120 days after the last session [42] and an 11–12% increase in skin hydration post-injections [41]. High-molecular-weight hyaluronic acid (HMW-HA), which predominates under homeostatic conditions, promotes CD44 receptor clustering and stabilizes interactions with ERM proteins and the actin cytoskeleton. In contrast, low-molecular-weight hyaluronic acid (LMW-HA), generated under pathological conditions through hyaluronidase activity and oxidative stress, disrupts CD44 clustering and increases receptor susceptibility to proteolytic cleavage. CD44 activation can be induced by platelet-derived growth factors or by hyaluronic acid fragments consisting of approximately 20–38 monomers. Upon binding to CD44 on the cell surface, hyaluronic acid regulates key cellular processes, including proliferation, differentiation, and migration, and contributes to the scavenging of reactive oxygen species. Additionally, CD44 signaling has been implicated in the regulation of apoptosis via mitochondria-dependent pathways [43,44,45,46].

A possible mechanism underlying the observed effects may be related to the strong hydrophilic properties of hyaluronic acid, which could improve hydration and elasticity of the oral mucosa, stabilize the mucosal moisture film, and reduce friction during oral functions. These changes may contribute to improved mucosal comfort and reduced irritation of sensory receptors, potentially influencing afferent sensory input involved in reflex salivary secretion [36]. Enhanced sensory feedback from the oral mucosa may therefore represent one hypothetical pathway contributing to improved salivary gland stimulation, particularly in the case of stimulated saliva flow. However, as no mechanistic parameters were directly assessed in the present study, these proposed explanations remain speculative and should be investigated in future studies.

Furthermore, in vivo studies have shown a positive effect of amino acids on atrophic changes in the salivary glands and salivary gland dysfunction in mice [15]. Owing to the similarity between skin and oral mucosa, we conducted a pilot study to investigate whether injections of a hyaluronic acid and amino acid solution could increase mucosal hydration and salivary secretion.

The present pilot study evaluated changes in salivary flow rate, salivary pH, buffering capacity, and patients’ perceived quality of life. In the intervention group, improvements were observed across all assessed parameters. An increase in salivary flow, normalization of salivary pH, enhancement of buffering capacity, and a positive change in patient-reported quality-of-life measures were noted during the follow-up visit. These findings suggest that the applied intervention may have a beneficial effect on both objective salivary parameters and subjective patient experience, which are key aspects in the management of xerostomia. Patients managed according to the study treatment protocol experienced no adverse events. No local or systemic complications were observed or reported during the treatment procedures or throughout the 8-week follow-up period in the intervention group. There were no reports of excessive or prolonged post-injection pain, persistent edema, bleeding, infection, allergic reactions, tissue necrosis, or other pathological changes in the oral tissues surrounding the injection sites. The injection procedure was generally well tolerated by all patients, and no participants discontinued treatment due to discomfort or treatment-related complications. Although patient acceptability of repeated injections was not formally assessed using a validated questionnaire, no participants expressed refusal or significant reluctance toward the treatment protocol. Nevertheless, given the pilot nature and limited sample size of this study, larger studies with formal safety monitoring and longer follow-up are needed to better characterize the tolerability, patient acceptance, and long-term safety profile of repeated intraoral applications of this therapy. In the control group, pH, saliva consistency, and buffering capacity did not change. Stimulated flow went up a bit (*p* = 0.002), but the median difference was 0 mL (95% CI: 0–0.5 mL). The intervention group, meanwhile, improved on every measure. That tiny shift in controls does not undermine our finding that the treatment works. If anything, it shows that spontaneous improvement cannot explain the size of the benefit we saw in treated patients.

The clear divergence in outcome trajectories between the two groups underscores the importance of including a non-intervention control group in studies assessing treatments for hyposalivation and xerostomia. While the present study was not powered to detect small between-group differences with high statistical certainty, the consistency of improvement in the intervention group alongside stability in the control group supports the plausibility of an intervention-related effect.

Given the pilot nature of the study, the absence of a placebo-controlled arm, and the relatively small sample size, these findings should be interpreted with caution. Additional limitations include the lack of long-term follow-up, which precluded assessment of the durability of the observed improvement in salivary secretion after treatment cessation, as well as the lack of blinding of the outcome assessor. Several evaluated parameters, including the mirror test, saliva consistency, and visual mucosal assessment, were partially subjective and therefore may have been influenced by observer expectation bias. Furthermore, the study relied on chairside diagnostic methods (Saliva-Check Buffer GC and VAS assessment), which were intentionally selected for their simplicity and ease of use in routine dental clinical practice, without comparison to reference-standard quantitative sialometry or validated xerostomia-specific patient-reported outcome instruments, which may limit precision and comparability with other studies. Another limitation is the heterogeneous etiology of dry mouth among included patients, including medication-related, age-associated, and postmenopausal xerostomia, which limits conclusions regarding the effectiveness of the intervention in specific patient subgroups.

Nevertheless, the results provide valuable preliminary data regarding feasibility, outcome variability, and potential effect size, which may support the design and formal sample size calculation of future adequately powered randomized controlled trials aimed at confirming the clinical effectiveness of the investigated intervention.

## 5. Conclusions

In this pilot study, intramucosal injections of HA and AA were associated with improvements in measured salivary parameters and in patient-reported dry mouth symptoms. These findings are hypothesis-generating and require confirmation in an adequately powered, placebo-controlled, blinded randomized trial. This research is short-term and requires further study. Because the use of HA and AAs in skin rejuvenation and hydration requires systematic repetition of treatments, we anticipate that the treatment conducted will also require continuation. Although clinical experience with similar applications suggests that repeated treatment may eventually be required, the optimal re-treatment interval cannot be determined from the present study and should be evaluated in future long-term follow-up studies.

## Figures and Tables

**Table 1:** Patient questionnaire.

Sex	Female				Male			
	
Age	≤29	30–39		40–49		50–59		≥60
				
Addiction	Smoking		Smoking in the past				Alcohol	
		
Medicines								
Dry mouth during meals	Yes				No			
	
Difficulty swallowing food	Yes				No			
	
Feeling of dry mouth during the day	Yes				No			
	
Feeling of dry mouth at night	Yes				No			
	
Feeling of dry mouth after waking up	Yes				No			
	
Difficulty sleeping due to dry mouth	Yes				No			
	
Waking up at night because of thirst	Yes				No			
	
Burning tongue	Yes				No			
	
Gum chewing to eliminate mouth dryness	Yes				No			
	
Other mucosa dryness	Yes				No			
	
VAS	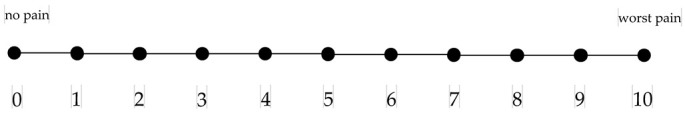							

**Table 2:** The study and control groups—divided by gender and age.

Variables	Study Group	Control Group
Age range	n (%)	n (%)
≤29	0 (0)	0 (0)
30–39	1 (2)	0 (0)
40–49	12 (24)	9 (18)
50–59	17 (34)	25 (50)
≥60	20 (40)	16 (32)
Gender		
Male	14 (28)	17 (34)
Female	36 (72)	33 (66)
Systemic disease	22 (44)	16 (32)

**Table 3:** Percentage distribution of symptoms before and after the study.

	Study Group		Control Group	
	Before	After	Before	After
symptoms	N (%)	N (%)	N (%)	N (%)
Dry mouth during meals	40 (80)	0 (0)	42 (84)	42 (84)
Difficulty swallowing food	46 (92)	0 (0)	43 (86)	43 (86)
Feeling of dry mouth during the day	42 (84)	0 (0)	40 (80)	40 (80)
Feeling of dry mouth at night	43 (86)	0 (0)	40 (80)	40 (80)
Feeling of dry mouth after waking up	46 (92)	5 (10)	45 (90)	45 (90)
Difficulty sleeping due to dry mouth	27 (54)	0 (0)	24 (48)	22 (44)
Waking up at night because of thirst	25 (50)	0 (0)	23 (46)	23 (46)
Burning tongue	35 (70)	3 (6)	30 (60)	30 (60)
Gum chewing to eliminate mouth dryness	14 (28)	0 (0)	14 (28)	9 (18)

**Table 4:** Pain intensity—VAS score.

		Pain Severity (VAS Grading)					
		No Pain (0)	Mild (1–3)	Moderate (4–6)	Severe (7–9)	Unbearable (10)	Total
		N (%)	N (%)	N (%)	N (%)	N (%)	
Study group	Before	0 (0)	0 (0)	26 (50)	22 (44)	3 (6)	50
	After	26 (52)	20 (40)	4 (8)	0 (0)	0 (0)	50
Control group	Before	0 (0)	3 (6)	26 (52)	20 (40)	1 (2)	50
	After	0 (0)	1 (2)	25 (50)	18 (36)	6 (12)	50

**Table 5:** Stimulated salivary flow—study group.

Variables	N	Mean	SD	Median	Min	Max	Kurtosis
Stimulated salivary flow after treatment	50	5.30	0.5345225	5.0	4	6	3.035714
Stimulated salivary flow before treatment	50	1.61	0.6490967	1.5	1	3	2.595901

**Table 6:** Stimulated saliva flow—study group.

Test	Statistic	*p*-Value	Estimate	CI_Low	CI_High
Wilcoxon Signed-Rank Test	1275	<0.001	3.75	3.50	4.00

**Table 7:** Stimulated Saliva Flow Directional Change Counts.

Change Direction	Number of Patients	Percentage (%)
Decrease	5	10
Increase	20	40
No change	25	50

**Table 8:** Normality test—simulated salivary flow before and after the research period.

Timepoint	Shapiro_Wilk_*p*	Anderson_Darling_*p*	Skewness	Kurtosis
SS_b4 SS_af	<0.001 0.0017	<0.001 <0.001	0.3951 0.0915	3.0027 2.4570

**Table 9:** Descriptive statistics—Salivary pH—study group.

Variable	N	Mean	SD	Median	Min	Max	Kurtosis	SE	95% CI
Saliva pH before treatment	50	6.54	0.43	6.49	5.62	7.52	−0.52	0.06	6.42 to 6.65
Saliva pH after treatment	50	7.31	0.33	7.31	6.43	8.05	0.05	0.05	7.22 to 7.40

**Table 10:** Paired *t*-test results—study group.

Test	Statistic (t)	*p*-Value	Mean Difference	95% CI
Paired *t*-test	−10.07	<0.001	−0.78	−0.93 to −0.62

**Table 11:** Descriptive statistics—Salivary pH—control group.

Variable	N	Mean	SD	Median	Min	Max	Kurtosis	SE	95% CI
Saliva pH beginning	50	6.10	0.31	6.0	5.2	6.8	3.27	0.04	6.02 to 6.19
Saliva pH after	50	6.14	0.36	6.2	5.2	6.8	3.58	0.05	6.04 to 6.25

**Table 12:** Saliva consistency distribution—study group.

Variable	N = 50 ^1^
Before Treatment	
Sticky saliva with increased viscosity	46 (92.0%)
Bubbly saliva with increased viscosity	4 (8.0%)
After Treatment	
Water saliva with normal viscosity	40 (80.0%)
Bubbly saliva with increased viscosity	10 (20.0%)

^1^ n (%).

**Table 13:** Saliva consistency transition following treatment.

Before	After: Water	After: Bubbly
Sticky	36	10
Bubbly	4	0

**Table 14:** Descriptive statistics—control group.

Measurement Period	Consistency	n	%
Before Treatment	SSIV	39	78
Before Treatment	BSIV	11	22
After Treatment	SSIV	39	78
After Treatment	BSIV	11	22

**Table 15:** Transition patterns—control group.

Before→After	SSIV	BSIV
SSIV	39	0
BSIV	0	11
Transition Pattern	Count	
Total patient pairs	50	
No change in consistency	50	
Changed consistency	0	
Percentage changed (%)	0	
Changed from SSIV to BSIV	0	
Changed from BSIV to SSIV	0	

**Table 16:** Saliva buffering capacity before and after treatment—study group.

Variables	n	Mean	sd	Median	Min	Max	95% CI_Lower	95% CI_Upper
Saliva buffering capacity before treatment	50	3.60	2.175935	4	0	6	3.00	4.20
Saliva buffering capacity after treatment	50	8.04	1.689825	8	4	10	7.57	8.51

**Table 17:** Saliva buffering capacity normality test—study group.

	W	*p*-Value
Saliva buffering capacity before treatment	0.926	0.004
Saliva buffering capacity after treatment	0.948	0.028

**Table 18:** Saliva buffering capacity—study group.

Statistical Measure	Value	Interpretation
Test Statistic (V)	76.5	
*p*-value	<0.001	Highly significant
Effect Size (r)	−0.83	Large effect [19] (Co)
Clinical Interpretation	Very large treatment effect	Clinically meaningful change

**Table 19:** Saliva buffering capacity before and after observation period—control group.

Salivary Buffering Capacity Measurement Period	n	Mean	SD	Median	IQR	Minimum	Maximum	Kurtosis
Before	50	4.72	1.443	4	2	0	6	3.768
After	50	4.72	1.604	4	2	2	8	2.476

**Table 20:** Normality test—control group.

Measurement Period	Shapiro–Wilk *p*	Anderson-Darling *p*
Salivary buffering capacity before treatment	<0.001	<0.001
Salivary buffering capacity after treatment	<0.001	<0.001

## Data Availability

The datasets used and analyzed during the current study are available from the corresponding author upon reasonable request.

## Supplementary Materials

The supporting information can be downloaded at https://www.mdpi.com/article/10.3390/life16060887/s1, Reference [47] is cited in the Supplementary Materials.
